# Aging Well in the Heart of America: Optimizing Housing Options Through Policy Reform

**DOI:** 10.1093/geroni/igaf122.1776

**Published:** 2025-12-31

**Authors:** Lana McKinney

**Affiliations:** University of Nebraska Omaha, Chicago, Illinois, United States

## Abstract

This research study investigates the growing housing availability and affordability challenges facing older adults in Kansas City, Missouri. Using the capability approach and the rational comprehensive model as theoretical frameworks, the study analyzes the current state of housing and proposes improvements to address availability and affordability concerns for older adults. A qualitative research design was employed, involving semi-structured interviews with 15 participants, including public officials, experts, and community members. Thematic analysis was used to analyze the interview transcripts, identifying recurring patterns and themes related to the participants’ perspectives on housing concepts, policies, and recommendations for improvement. The analysis revealed four key themes: affordability and availability deprivation, policy gaps, the need for targeted capital resources and rational decision-making, and potential policy solutions. The findings highlight a critical gap between existing housing policies and the needs of older adults, underscoring the necessity for increased affordability and availability of housing options. The study proposes several policy recommendations, including streamlining the regulatory process, enhancing public and private partnerships, directed capital for housing from local, state, and federal government, using implementation science to enact policy. These recommendations aim to address the identified challenges and ignite policy reform on behalf of older adults providing suitable and supportive housing.

